# Supplementary material for: A fault-intrusion-tolerant system and deadline-aware algorithm for scheduling scientific workflow in the cloud
Source: PeerJ Comput Sci. 2021 Nov 2;7:e747. doi: 10.7717/peerj-cs.747 (PMC8576554; doi:10.7717/peerj-cs.747)
Supplement: Supplemental Information 6 [file peerj-cs-07-747-s006.zip › Myproject1.1/docs/org/cloudbus/cloudsim/core/predicates/PredicateFrom.html]

PredicateFrom


JavaScript is disabled on your browser.


- Overview
- Package
- Class
- Use
- Tree
- Deprecated
- Index
- Help

- Prev Class
- Next Class

- Frames
- No Frames

- All Classes

- Summary:
- Nested |
- Field |
- Constr |
- Method

- Detail:
- Field |
- Constr |
- Method


org.cloudbus.cloudsim.core.predicates

## Class PredicateFrom

- java.lang.Object
- - org.cloudbus.cloudsim.core.predicates.Predicate
  - - org.cloudbus.cloudsim.core.predicates.PredicateFrom

- ---

    

  ```
  public class PredicateFrom
  extends Predicate
  ```

  A predicate which selects events from specific entities.  
  The idea of simulation predicates was copied from SimJava 2.

  Since:
  :   CloudSim Toolkit 1.0

  See Also:
  :   `PredicateNotFrom`,
      `Predicate`

- - ### Constructor Summary

    Constructors

    Constructor and Description

    `PredicateFrom(int sourceId)`

    Constructor used to select events that were sent by a specific entity.

    `PredicateFrom(int[] sourceIds)`

    Constructor used to select events that were sent by any entity from a given set.
  - ### Method Summary

    Methods

    | Modifier and Type | Method and Description |
    | --- | --- |
    | `boolean` | `match(SimEvent ev)` The match function called by `Simulation`, not used directly by the user. |

    - ### Methods inherited from class java.lang.Object

      `clone, equals, finalize, getClass, hashCode, notify, notifyAll, toString, wait, wait, wait`

- - ### Constructor Detail


    - #### PredicateFrom

      ```
      public PredicateFrom(int sourceId)
      ```

      Constructor used to select events that were sent by a specific entity.

      Parameters:
      :   `sourceId` - the id number of the source entity


    - #### PredicateFrom

      ```
      public PredicateFrom(int[] sourceIds)
      ```

      Constructor used to select events that were sent by any entity from a given set.

      Parameters:
      :   `sourceIds` - the set of id numbers of the source entities
  - ### Method Detail


    - #### match

      ```
      public boolean match(SimEvent ev)
      ```

      The match function called by `Simulation`, not used directly by the user.

      **Specified by:**
      :   `match` in class `Predicate`

      Parameters:
      :   `ev` - the event to check

      Returns:
      :   `true` if the event matches the predicate, `false` otherwise


- Overview
- Package
- Class
- Use
- Tree
- Deprecated
- Index
- Help

- Prev Class
- Next Class

- Frames
- No Frames

- All Classes

- Summary:
- Nested |
- Field |
- Constr |
- Method

- Detail:
- Field |
- Constr |
- Method
